# Organização dos sistemas locais de saúde em municípios rurais remotos brasileiros no enfrentamento da pandemia de COVID-19

**DOI:** 10.1590/0102-311XPT170723

**Published:** 2024-07-29

**Authors:** Nereide Lucia Martinelli, Simone Schenkman, Elisete Duarte, Cleide Lavieri Martins, Renata Elisie Barbalho, Márcia Cristina Rodrigues Fausto, Aylene Emilia Moraes Bousquat

**Affiliations:** 1 Instituto de Saúde Coletiva, Universidade Federal de Mato Grosso, Cuiabá, Brasil.; 2 Faculdade de Saúde Pública, Universidade de São Paulo, São Paulo, Brasil.; 3 Escola Nacional de Saúde Pública Sergio Arouca, Fundação Oswaldo Cruz, Rio de Janeiro, Brasil.

**Keywords:** Atenção Primária à Saúde, Acesso aos Serviços de Saúde, Sistemas Locais de Saúde, Primary Health Care, Access to Health Services, Local Health Systems, Atención Primaria de Salud, Acceso a los Servicios de Salud, Sistemas Locales de Salud

## Abstract

Na pandemia de COVID-19, as populações que vivem mais afastadas dos centros urbanos enfrentaram imensas dificuldades no acesso aos serviços de saúde. O objetivo deste estudo é analisar como os municípios rurais remotos brasileiros enfrentaram a pandemia de COVID-19, tendo como base sua resposta política, estrutural e organizativa ao acesso à saúde. Trata-se de estudo qualitativo de casos múltiplos com a análise de conteúdo temática e dedutiva de 51 entrevistas conduzidas com gestores e profissionais de saúde em 16 municípios rurais remotos dos estados de Rondônia, Mato Grosso, Tocantins, Piauí, Minas Gerais e Amazonas. Com dinâmicas socioespaciais próprias, grandes distâncias até os centros de referência, os municípios rurais remotos responderam às demandas da pandemia, mas não tiveram suas necessidades atendidas oportunamente. Preservaram a comunicação com a população, reorganizaram o sistema local centrado na atenção primária à saúde (APS), alteraram o funcionamento das unidades de saúde, ultrapassando os limites de suas atribuições para prestar o cuidado necessário e aguardar o encaminhamento aos demais níveis de complexidade. Enfrentaram a escassez de serviços, as lacunas assistenciais da rede regional e o transporte sanitário inadequado. A pandemia reiterou as dificuldades da APS em coordenar o cuidado e expôs os vazios assistenciais nas regiões de referência. A provisão equitativa e resolutiva do sistema local de saúde nos municípios rurais remotos implica na articulação interfederativa à formulação e implementação de políticas públicas de modo a assegurar o direito à saúde.

## Abstract (en)

During the COVID-19 pandemic, populations living further away from urban centers faced immense difficulties accessing health services. This study aims to analyze how Brazilian remote rural municipalities faced the COVID-19 pandemic based on their political, structural, and organizational response to access to healthcare. A qualitative study of multiple cases was conducted with thematic and deductive content analysis of 51 interviews conducted with managers and healthcare professionals in 16 remote rural municipalities in the states of Rondônia, Mato Grosso, Tocantins, Piauí, Minas Gerais, and Amazonas. With their socio-spatial dynamics and long distances to reference centers, the remote rural municipalities responded to the demands of the pandemic but did not have their needs met promptly. They preserved communication with the population, reorganized the local system centered on primary health care (PHC), and changed the functioning of healthcare units, exceeding the limits of their responsibilities to provide the necessary care and awaiting referral to other levels of complexity. They faced a shortage of services, gaps in assistance in the regional network, and inadequate healthcare transport. The pandemic reiterated PHC’s difficulties in coordinating care, exposing care gaps in reference regions. The equitable and resolute provision of the local health system in the remote rural municipalities implies inter-federative articulation in formulating and implementing public policies to ensure the right to health.

## Resumen

En la pandemia de COVID-19, las poblaciones que viven más alejadas de los centros urbanos enfrentaron inmensas dificultades para acceder a los servicios de salud. El objetivo de este estudio es analizar cómo los municipios rurales remotos de Brasil enfrentaron la pandemia de COVID-19, a partir de su respuesta política, estructural y organizativa al acceso a la salud. Se realizó el estudio cualitativo de casos múltiple con el análisis de contenido temático y deductivo de 51 entrevistas realizadas con gestores y profesionales de la salud en 16 municipios rurales remotos de los estados de Rondônia, Mato Grosso, Tocantins, Piauí, Minas Gerais y Amazonas. Con dinámicas socioespaciales propias y alejados de los centros de referencia, los municipios rurales remotos respondieron a las demandas de la pandemia, pero no se atendieron sus necesidades de manera oportuna. Preservaron la comunicación con la población, reorganizaron el sistema local centrado en la atención primaria de salud (APS), modificaron el funcionamiento de las unidades de salud, superando los límites de sus atribuciones para proporcionar la atención necesaria, y esperar la derivación a los demás niveles de complejidad. Enfrentaron la falta de servicios, las lagunas asistenciales de la red regional y el transporte sanitario inadecuado. La pandemia reafirmó las dificultades de la APS para coordinar la atención y expuso las lagunas asistenciales en las regiones de referencia. La provisión equitativa y resolutiva del sistema de salud local en los municipios rurales remotos implica en la articulación interfederativa para elaborar e implementar políticas públicas para asegurar el derecho a la salud.

## Introdução

Na pandemia de COVID-19, as populações mais afastadas dos centros urbanos enfrentaram dificuldades no acesso aos serviços de saúde, expressando desigualdades na oferta de serviços e disponibilidade de profissionais. Esses vazios assistenciais demandam dos gestores municipais brasileiros o estabelecimento de parcerias para reorganizar os sistemas locais de saúde, especialmente na atenção especializada ^1^ ^,^ ^2^ ^,^ ^3^ ^,^ ^4^ ^,^ ^5^.

Os efeitos causados pelo SARS-CoV-2 afetaram diferentes lugares, a incidência da COVID-19 variou e provocou mudanças em todos os níveis do sistema de saúde, alterando a rotina e a dinâmica dos territórios ^6^ ^,^ ^7^. No contexto de uma pandemia, declarada pela Organização Mundial da Saúde (OMS) no dia 11 de março de 2020, e na perspectiva de uma atenção primária à saúde (APS) resolutiva e ampliada, a organização e a disponibilidade de serviços de saúde locais tornam-se ainda mais relevantes, variando em função do tipo de governança, de gestão, do modelo de cuidado e das necessidades da população ^1^ ^,^ ^8^.

Entre as populações vulneráveis, estão aquelas que vivem nos 323 municípios rurais remotos no Brasil ^9^. Com populações dispersas, enfrentam maiores dificuldades pela distância até os centros urbanos que concentram importante parte dos recursos de saúde ^1^ ^,^ ^8^ ^,^ ^9^.

Independentemente da crise epidemiológica e sanitária, a necessidade de primeiro contato com o serviço de saúde foi reiterada por diversos autores ^10^ ^,^ ^11^. A APS enfrentou novos desafios pelo seu potencial para intervir e comunicar-se com a comunidade, gerenciar os riscos, realizar a vigilância, conter a disseminação do vírus, e prestar os primeiros cuidados demandados pela COVID-19 ^10^ ^,^ ^12^.

Nos municípios rurais remotos, a rede de atenção é constituída basicamente por serviços de APS, a principal porta de entrada ao Sistema Único de Saúde (SUS). É desenvolvida pelas equipes de atenção básica e, especialmente, da Estratégia Saúde da Família (ESF) ^11^ ^,^ ^13^ ^,^ ^14^, mas tem escassez de profissionais, de recursos financeiros ^6^ e maior dependência da rede de serviços de referência localizadas nas regiões e em cidades com maior dinamismo e poder econômico ^6^ ^,^ ^15^ ^,^ ^16^ ^,^ ^17^.

As dificuldades dos municípios rurais remotos para suprir as necessidades da população, garantir o transporte adequado e o acesso aos serviços de saúde em tempo hábil ^1^ ^,^ ^8^ ^,^ ^11^ também são encontradas nos Estados Unidos, Austrália, Canadá, China, Índia, Portugal, entre outros. Resultam em desigualdades de acesso entre populações rurais e urbanas, impõem desafios para organizar os serviços de saúde e assegurar o acesso da população em tempo oportuno ^15^ ^,^ ^16^ ^,^ ^17^ ^,^ ^18^ ^,^ ^19^ ^,^ ^20^.

É partindo desse cenário, com dificuldades e desigualdades locais e regionais na oferta e distribuição dos serviços, que os sistemas locais de saúde dos municípios rurais remotos tiveram que enfrentar a maior crise sanitária dos últimos 100 anos ^3^ ^,^ ^8^ ^,^ ^10^. O objetivo deste estudo é analisar como os municípios rurais remotos brasileiros enfrentaram a pandemia de COVID-19, tendo como base sua resposta política, estrutural e organizativa ao acesso à saúde.

## Método

Estudo qualitativo de casos múltiplos, integrante da pesquisa nacional *Desafios e Estratégias no Enfrentamento da Pandemia da COVID-19 nos Municípios Rurais e Remotos Brasileiros*. O enfoque metodológico apoiou-se no instrumental de políticas públicas ^21^ ^,^ ^22^, desdobrado na análise multidimensional que analisou os condicionantes políticos, estruturais e organizativos, para compreender como se deu a reorganização do sistema de saúde locorregional nos municípios rurais remotos durante a pandemia da COVID-19.

Inicialmente, em 2020, foi realizada a coleta de dados secundários nos bancos de dados oficiais: Instituto Brasileiro de Geografia e Estatística (IBGE), o Departamento de Informática do SUS (DATASUS), Programa das Nações Unidas para o Desenvolvimento (PNUD; 2010); Cadastro Nacional de Estabelecimentos de Saúde (CNES; 2019). Paralelamente, realizou-se busca na internet de documentos, notícias, planos de contingência, entre outros, que permitissem identificar as atividades realizadas no combate à pandemia em todos os municípios rurais remotos brasileiros. Foi definido que no mínimo dez documentos seriam necessários para essa análise. Dos 323 municípios rurais remotos, apenas 26 não cumpriram esse critério. Em 2022, foram coletados os recursos financeiros transferidos por meio de emendas parlamentares, entre 2020 e 2022 (do Fundo Nacional de Saúde/Ministério da Saúde) e o valor destinado ao enfrentamento da COVID-19.

Foi criado um escore de enfrentamento à COVID-19 a partir da análise documental, sendo definidas 29 variáveis distribuídas em seis dimensões: condução política (plano, decretos, comitê de enfrentamento); organização dos serviços (fluxos de atendimento, unidades de referência, atendimento remoto/rotina); isolamento social (suspensão das atividades); vigilância de casos (monitoramento de casos/contactantes); vigilância de fronteiras (barreiras, quarentena); e apoio social (máscaras, produtos de higiene, merenda, cestas básicas, suspensão de cobrança de contas). Os pesos foram iguais para as dimensões e suas variáveis componentes. As variáveis e dimensões variaram de 0 a 1, sendo 1 o mais completo.

Entre os que obtiveram os escores com maior e menor completude, selecionaram-se 16 municípios rurais remotos para realizar entrevistas: Campinápolis, Figueirópolis D’Oeste, Indiavaí, Nova Canaã do Norte, Nova Lacerda, Serra Nova Dourada, Terra Nova do Norte e Vila Bela da Santíssima Trindade (Mato Grosso); Campos Lindos e Goianorte (Tocantins); Indaiabira e Rubelita (Minas Gerais); Maués (Amazonas); Pajeú do Piauí e Porto Alegre do Piauí (Piauí); e São Miguel do Guaporé (Rondônia).

As entrevistas foram apoiadas por roteiros semiestruturados, embasadas no instrumental de análise de políticas públicas ^21^ ^,^ ^22^ e categoria acesso, como referencial teórico ^23^. Analisaram-se as dimensões: (1) política - disputas entre níveis de governo e o poder decisório; (2) estrutural - a capacidade instalada laboratorial, ambulatorial, hospitalar e de leitos de unidade de tratamento intensivo (UTI), aquisição dos insumos específicos, equipamentos, incluindo os equipamentos de proteção individual (EPI); e (3) organizacional - estratégias priorizadas, acompanhamento e atendimento remotos, acesso aos serviços da rede local e regionalizada, mecanismos de regulação, redesenho dos fluxos e reorganização das ações e serviços da APS.

Utilizaram-se roteiros diferenciados para os distintos atores políticos: secretários municipais de saúde (n = 13), coordenadores da atenção básica (n = 12), coordenadores de vigilância epidemiológica/em saúde (n = 9), profissionais da ESF (n = 10), prefeitos (n = 2), representantes do Conselho Municipal de Saúde (CMS) (n = 6), responsáveis pela regulação (n = 2) e assistentes sociais (n = 3). Foram realizadas 47 entrevistas virtuais individuais e sete conjuntas, resultando em 59 entrevistados, entre julho de 2021 e abril de 2022, por meio das plataformas Meet (https://meet.google.com/) e Zoom (https://zoom.us/pt), todas gravadas. Quatro entrevistas previstas não foram feitas nesse primeiro momento, por problemas de agenda, mas foram realizadas presencialmente em 2022. No total, foram 51 entrevistas com 63 entrevistados.

Foram entrevistadas 43 mulheres (68%) e 20 homens (32%); com média de idade de 37 anos, com desvio padrão de oito anos (23-62 anos); mediana do tempo de atuação no município de nove anos (0,33-31 anos), com dois anos (0,03-24 anos) na função atual; 22% apresentavam Ensino Médio completo, nível técnico ou Superior incompleto; 63% com Superior completo e 15% com Especialização/Pós-graduação.

A análise de conteúdo temática e dedutiva, utilizou roteiro previamente definido que contemplou as dimensões e categorias de análise (Quadro 1), a possibilidade de captar novos conteúdos e as categorias no momento analítico, reservando um campo para inclusão dos trechos das falas dos entrevistados.

**Quadro 1:** Dimensões e categorias de análise consideradas para as entrevistas. Municípios rurais remotos brasileiros, 2021-2022.

DIMENSÕES	CATEGORIAS DE ANÁLISE
Política	1. Plano de contingência 2. Comitê de enfrentamento: funcionamento e representantes 3. Orientação por decretos: condução da gestão local 4. Articulação: gestão, decretos e planos; relação entre os níveis de governo; consórcios intermunicipais
Estrutura	5. Insumos e serviços críticos: equipamentos, medicamentos, aquisição de EPI, cooperação intermunicipal 6. Capacidade instalada ambulatorial, hospitalar e de leitos de UTI 7. Acesso à UBS na pandemia-transporte específico 8. SADT e assistência farmacêutica-referência local para exames e entrega de medicamentos 9. Transporte (intermunicipal) equipado, específico para COVID-19
Organização	10. Fluxo inicial dos casos e atendimento de rotina 11. Unidade de referência local para COVID-19 12. Articulação com a rede (NASF), saúde bucal, reabilitação, apoio psicológico, hospitais e laboratórios 13. Regulação, fluxo de referência e intercorrências na espera por um leito de UTI ou para exames de imagem

EPI: equipamentos de proteção individual; UBS: unidade básica de saúde; UTI: unidade de tratamento intensivo; SADT: Serviço de Apoio Diagnóstico Terapêutico; NASF: Núcleo de Apoio à Saúde da Família.

Os roteiros de análise foram submetidos a pré-testes, ajustados por dois pesquisadores e, finalmente, consolidados por cinco pesquisadores com perfis diferentes, que analisaram as entrevistas de cinco municípios. Uma dupla de pesquisadores, ao concluir a análise de todas as entrevistas, realizou a síntese global do município de forma combinada às falas dos entrevistados. O posicionamento dos atores-chave nas entrevistas permitiu entender as relações formais e informais estabelecidas entre entes federativos, gestores, prestadores de serviços, profissionais e população para intervir e viabilizar o acesso à assistência à saúde durante a pandemia.

A pesquisa foi aprovada pelo Comitê de Ética da Faculdade de Saúde Pública da Universidade de São Paulo (CAAE 37672620.8.0000.5421, parecer nº 4.285.824) e todos os entrevistados assinaram o Termo de Consentimento Livre e Esclarecido (TCLE).

Os resultados serão apresentados segundo as dimensões de análise priorizadas. Elas apresentam interseções importantes, sobretudo no contexto pandêmico que exigiu mudanças e aproximações nas várias dimensões, borrando os limites entre si.

## Resultados

Os 323 municípios rurais remotos brasileiros concentram-se nas regiões Norte, Nordeste e Centro-oeste ^9^. Os 16 municípios rurais remotos analisados são de pequeno porte, a maioria de baixa densidade demográfica e menos de 10 mil habitantes. Estão localizados nos seguintes estados: Rondônia, Mato Grosso, Tocantins, Piauí, Minas Gerais e Amazonas (Tabela 1).

**Tabela 1:** Municípios rurais remotos selecionados, segundo informações sociodemográficas e financeiras. Brasil, 2019.

Municípios	População	Área (km^2^)	Densidade demográfica	**PIB *per capita* (R$)**	Bolsa Família (%)	IDHM	Índice de Gini	Emendas parlamentares (R$)	Emendas parlamentares (COVID-19) (R$)
Serra Nova Dourada	1.650	1.490,7	1.01	12.359	44,58	Médio	0,46	499.881,00	-
Indiavaí	2.752	592,5	4,22	13.307	14,29	Médio	0,48	793.556,00	-
Figueirópolis D’Oeste	3.494	891,4	3,95	13.443	8,09	Médio	0,43	1.676.449,00	-
Nova Lacerda	6.640	4.780,4	1,28	20.447	29,10	Médio	0,53	1.449.887,00	-
Terra Nova do Norte	9.667	2.399,7	3,74	16.661	20,11	Médio	0,50	4.728.717,00	-
Nova Canaã do Norte	12.787	5.953,1	2,07	25.332	10,02	Médio	0,57	4.347.236,00	-
Campinápolis	15.980	5.978,9	2,59	10.525	24,71	Baixo	0,69	3.507.478,00	-
Vila Bela da Santísima Trindade	16.128	13.443,6	1,14	21.813	22,06	Médio	0,59	4.122.852,07	-
São Miguel do Guaporé	23.005	7.815,0	2,99	16.701	14,05	Médio	0,63	7.942.534,00	100.000,00
Maués	63.905	39.988,0	1,50	6.272	54,67	Baixo	0,64	53.860.959,00	19.790.272,00
Rubelita	5.995	1.110,2	6,39	5.598	48,63	Baixo	0,50	2.038.023,00	-
Indaiabira	7.351	1.004,1	7,50	6.650	46,73	Médio	0,48	4.119.200,00	-
Porto Alegre do Piauí	2.710	1.137,0	2,26	7.853	54,70	Baixo	0,48	1.761.638,00	250.000,00
Pajeú do Piauí	3.389	1.075,0	3,07	5.059	62,08	Baixo	0,59	2.258.450,00	200.000,00
Goianorte	5.123	1.801,0	2,85	9.093	56,38	Médio	0,54	3.267.138,00	200.000,00
Campos Lindos	10.116	3.240,0	2,90	31.756	26,52	Baixo	0,68	3.882.456,00	-

IDHM: Índice de Desenvolvimento Humano Municipal; PIB: produto interno bruto.

Fonte: Programa das Nações Unidas para o Desenvolvimento ^39^; Instituto Brasileiro de Geografia e Estatística ^40^; Tribunal de Contas da União ^41^; Ministério do Desenvolvimento Social ^42^; Fundo Nacional de Saúde ^43^.

Na caracterização socioeconômica em 2019, o produto interno bruto (PIB) *per capita* variou de R$ 5.059,00 em Pajeú do Piauí a R$ 31.756,00 em Campos Lindos, sendo que 62,08% e 26,52% da população, respectivamente, recebe Bolsa Família, ambos com Índice de Desenvolvimento Humano Municipal (IDHM) baixo. Campos Lindos tem o maior PIB, e, dos seis municípios rurais remotos que apresentam IDHM baixo, dois têm PIB *per capita* maior que R$ 10.000,00. Os demais municípios apresentam IDHM médio (Tabela 1).

A transferência de recursos via emendas parlamentares totalizou entre os 16 municípios rurais remotos mais de R$ 100 milhões no período de 2020 a 2022 e os que mais receberam foram Maués (54%) e, entre o restante (46%), São Miguel do Guaporé (17%), Terra Nova do Norte (10%), Nova Canaã (9%), Vila Bela da Santíssima Trindade (9%), Indaiabira (9%), Campos Lindos (8%), Campinápolis (8%) e Goianorte (7%) (Tabela 1).

### Política

Os sistemas locais de saúde nos municípios rurais remotos foram reorganizados com base nos decretos dos governos federal e estadual. Orientaram os gestores para elaborar os decretos municipais, o plano de contingência e criar os comitês de enfrentamento. Poucos utilizaram o plano de contingência como instrumento estratégico à condução política local, adotando características mais regionais. Assim, não foi reconhecido como delineador da condução local, apresentando poucos relatos.

Na articulação entre os níveis de governo, houve diversidade locorregional. O apoio estadual ocorreu por meio das instâncias regionais, da definição dos fluxos de referências regional e macrorregional, da regulação do transporte aéreo, da instalação de leitos de UTI e de hospitais de campanha, que serão detalhados nas respectivas dimensões. Ocorreram situações em que o nível local teve de se expandir para compensar a ausência e/ou a baixa capacidade instalada dos serviços de referência. Poucos municípios rurais remotos participaram de Consórcios Intermunicipais de Saúde (CIS) como estratégia para ampliar o acesso.

A cooperação técnica das instâncias regionais, como a Comissão de Intergestores Regional (CIR) contribuíram para a tomada de decisões e condução das medidas de contingência: “*as reuniões de CIR foi muito importante...*” (gestor; município 4). Por meio da CIR, os gestores se sentiram apoiados “*o escritório... fazia os encontros... por ‘live’, toda quarta-feira... faziam sugestões,... os apontamentos... o que era possível... era adotado*” (profissional da ESF/coordenador da atenção básica - enfermeiro/a; município 3). Reconhecidamente, propiciaram a articulação intermunicipal e interfederativa e estimularam a troca de experiências “*...a troca de experiência é muito válida...*” (gestor; município 4).

A criação dos comitês de enfrentamento nem sempre ocorreu de forma espontânea; em um município, resultou da indução do Ministério Público: “*a iniciativa de reunir várias esferas da sociedade, pautar... decidir o rumo das coisas conjuntamente foi do Ministério Público*” (profissional da ESF - enfermeiro/a; município 10). Apesar da diferença nos processos, foram criados nos 16 municípios rurais remotos, constituídos por atores do setor público, privado e representações da população: prefeito, secretário da saúde, polícia municipal, comércio, conselheiros de saúde, igreja católica e/ou evangélica, secretaria de educação e de promoção social, promotoria pública e profissionais da saúde. A participação dos profissionais, em alguns municípios rurais remotos, contribuiu para promover medidas de contingenciamento antes da ocorrência do primeiro caso.

Os comitês foram consultivos e essenciais para elaborar os planos de contingência, mobilizar e organizar a rede local “*eram vários... representantes, não era deliberativo, a decisão final era do executivo..., todo mundo dava as suas opiniões, mas quem optava pelo decreto se ia fechar e o que não ia fechar, na verdade era o prefeito*” (profissional da ESF - enfermeiro/a; município 9). Além disso, foram determinantes para definir e aplicar as medidas preventivas e sanitárias. Os entrevistados afirmaram realizar o chamamento das representações “*...nós verificamos quem seriam as instituições que poderiam agregar... e solicitamos os representantes, o critério... foi esse*” (gestor; município 10), mas nem todos contaram com a participação social.

Nas reuniões dos comitês, havia conflitos; sobretudo na primeira onda, ocorreram pela resistência dos estabelecimentos em acatar as medidas restritivas. “*Vivia... uma guerra,... público contra privado, tipo, ah vocês são públicos, vocês estão com o salário garantido, não precisam abrir nada, vocês querem mais é que feche tudo, e aí quando a gente tinha essas reuniões... começava a ser muito agredido,...como se nós estivéssemos querendo prejudicar o andamento do município…*” (profissional da ESF - enfermeiro/a; município 9).

A maioria dos municípios rurais remotos relatou a relevância dos decretos federais e estaduais e a experiência positiva com o comitê de enfrentamento, norteador da condução política, que concretizou a articulação e participação de outros setores e da população. Poucos reportaram conflitos nas deliberações, dificuldades extremas na adesão às medidas de contingenciamento, ausência de participação de alguns setores e participação local.

Na dimensão política, identificaram-se diversidades nas relações intergovernamentais, pouca participação interfederativa nas decisões, apoio técnico das instâncias regionais das Secretarias de Estado da Saúde (SES), em algumas regiões, e uso dos CIS como estratégia de ampliação da rede de atenção.

### Estrutura

A reorganização da rede de atenção variou e foi ampliada com a instalação de centros de COVID-19, aquisição de equipamentos e serviços, mas continuou limitada, principalmente no início, pela escassez de recursos e insumos e pela baixa capacidade instalada local. As equipes de atenção básica e da ESF viabilizaram o primeiro contato do usuário, mas nem todos os municípios rurais remotos, apresentavam cobertura de 100% de APS (Tabela 2).

**Tabela 2:** Municípios rurais remotos brasileiros, segundo Unidade Federativa (UF), região de saúde, rede de atenção local e cobertura de atenção primária à saúde (APS) e de agentes comunitários de saúde (ACS), em 2019.

Municípios	UF	Região de saúde	Rede de atenção local			Cobertura de APS e de ACS (%)		
			APS	CAPS/NASF/Centro de Reabilitação	Hospital	Equipes da ESF	Equipes da atenção básica	ACS
Serra Nova Dourada	Mato Grosso	Norte Araguaia Karajá	1	1	-	100	100	100
Indiavaí	Mato Grosso	Oeste Matogrossense	1	1	-	100	100	100
Figueirópolis D’Oeste	Mato Grosso	Sudoeste Matogrossense	1	2	-	98	98	100
Nova Lacerda	Mato Grosso	Sudoeste Matogrossense	3	2	-	100	100	100
Terra Nova do Norte	Mato Grosso	Vale do Peixoto	5	1	-	100	100	100
Nova Canaã do Norte	Mato Grosso	Norte Matogrossense	7	1	-	81	81	99
Campinápolis	Mato Grosso	Garças Araguaia	3	1	1	65	65	100
Vila Bela da Santísima Trindade	Mato Grosso	Sudoeste Matogrossense	13	1		100	100	100
São Miguel do Guaporé	Rondônia	Central	5	2	1	45	61	65
Maués	Amazonas	Baixo Amazonas	8	3	1	93	93	100
Rubelita	Minas Gerais	Salinas	6	1	-	100	100	100
Indaiabira	Minas Gerais	Taiobeiras	7	-	-	100	100	100
Porto Alegre do Piauí	Piauí	Vale dos Rios Piaui e Itaueira	2	-	1 *	100	100	100
Pajeú do Piauí	Piauí	Vale dos Rios Piaui e Itaueira	7	-	-	100	100	100
Goianorte	Tocantins	Cerrado Tocantins Araguaia	2	-	-	100	100	100
Campos Lindos	Tocantins	Médio Norte Araguaia	1	-	-	70	70	100

CAPS: Centros de Apoio Psicossocial; ESF: Estratégia Saúde da Família; NASF: Núcleos de Apoio à Saúde da Família.

Fonte: elaboração própria com base nos dados do Instituto Brasileiro de Geografia e Estatística ^44^; Departamento de Informática do SUS ^45^; Cadastro Nacional dos Estabelecimentos de Saúde ^46^.

* Unidade mista.

Durante a pandemia, alguns municípios rurais remotos, em espaços distintos ou nos mesmos locais das unidades básicas de saúde (UBS), instalaram centros de COVID-19 com leitos de observação (Tabela 2). Sete municípios rurais remotos dispõem de unidade de Serviços de Apoio Diagnóstico Terapêutico (SADT) isolada; em outros, há apenas unidades de coleta. Pela falta de capacidade instalada local e regulada pelo estado, as demandas por serviços de exame laboratorial e de imagem (tomografia computadorizada - TC), consultas especializadas, hospitais gerais e UTI eram encaminhadas às regiões e macrorregiões. Também foram liberadas pelos CIS ou pela compra municipal de serviços privados, mas com custos e desembolso direto pelo usuário, “*a população tinha que pagar as TC,... muita dificuldade,... a gente cobrou para que a Secretaria pagasse as TCs..., as ressonâncias... para a COVID...*” (representante do CMS; município 7).

Em algumas regiões, a estrutura da rede de atenção anterior à pandemia era incompatível à instalação de leitos de UTI, no setor público e privado, a rede de atenção nas regiões de saúde sofre com a falta de serviços, até mesmo no setor privado. Durante a pandemia, alguns municípios rurais remotos compensaram esses problemas com pontos adicionais à rede e instalação de leitos de UTI no setor privado, “*...o estado nos ajudou... UTI... conseguimos dez leitos.... foi um sonho... a região... precisava*” (gestor; município 5).

A expansão da capacidade laboratorial local, a instalação dos hospitais de campanha e os CIS de Mato Grosso e Rondônia, foram iniciativas municipais e alternativas para ampliar o escopo e amenizar a insuficiência de serviços locorregionais, agilizando o acesso à rede de saúde: “*o consórcio ajudou na compra das tomografias, dos EPIs, testes rápidos...compramos tudo isso via consórcio*” (gestor; município 2).

Os custos aumentaram, as despesas diversificaram-se e as transferências de recursos financeiros fundo a fundo, ou por meio das emendas parlamentares, contribuíram para contornar as dificuldades. A maioria relatou que os recursos foram utilizados para contratar e ou contratualizar exames laboratoriais, ampliar as cotas de tomografia, instalar os centros de COVID-19 e realizar a compra de ambulâncias, de insumos e EPI “*facilitou bastante... conseguir emenda parlamentar*” (gestor; município 10). Na primeira onda da pandemia, as máscaras e aventais disponibilizados aos profissionais foram confeccionados pela assistência social, e o álcool em gel doado (por empresas e outros setores) contribuiu para que houvesse suficiência.

Os meios de transporte variaram conforme a disponibilidade, as necessidades, o tipo e a distância ao local de referência (Quadro 2). Em Maués, foi utilizado o transporte aéreo e as *ambulanchas* (ambulância fluvial), os demais faziam o uso de ambulâncias básicas ou equipadas com semi-UTI. Alguns municípios rurais remotos tiveram dificuldade para contratar e/ou adquirir semi-UTI e, quando conseguiam, os custos eram altos. Além disso, a falta de manutenção dos equipamentos, sua inadequação e o excesso de deslocamentos dificultaram a gestão local, com pouco apoio estadual.

**Quadro 2:** Municípios rurais remotos, municípios de referência regional, distância e tipo de transporte sanitário utilizado durante a pandemia. Brasil, 2021-2022.

MUNICÍPIOS	UF	MUNICÍPIO DE REFERÊNCIA NA REGIÃO E DISTÂNCIA (EM KM)	TRANSPORTE SANITÁRIO
Serra Nova Dourada	Mato Grosso	São Félix do Araguaia (146,8)	Ambulâncias e semi-UTI
Indiavaí	Mato Grosso	Cáceres (153,8)	Ambulâncias
Figueirópolis D’Oeste	Mato Grosso	Pontes e Lacerda (80,7)	Ambulâncias e semi-UTI
Nova Lacerda	Mato Grosso	Pontes e Lacerda (99,5)	Ambulâncias e semi-UTI
Terra Nova do Norte	Mato Grosso	Peixoto de Azevedo (621,8)	Ambulâncias
Nova Canaã do Norte	Mato Grosso	Colíder (49,7)	Ambulâncias
Campinápolis	Mato Grosso	Barra do Garças (232)	Ambulância (1)
Vila Bela da Santísima Trindade	Mato Grosso	Pontes e Lacerda (77,2)	Ambulâncias, micro-ônibus e mini-UTI
São Miguel do Guaporé	Rondônia	Rolim de Moura (111,9); Cacoal (177); Ji-Paraná (112)	Ambulâncias
Maués	Amazonas	Manaus (612)	Aéreo e *Ambulanchas*
Rubelita	Minas Gerais	Salinas (37); Montes Claros (249)	Ambulâncias
Indaiabira	Minas Gerais	Taiobeiras (45); Montes Claros (307)	Ambulâncias
Porto Alegre do Piauí	Piauí	Floriano (162)	Ambulâncias
Pajeú do Piauí	Piauí	Floriano (167); Canto de Buritis (37)	Ambulâncias
Goianorte	Tocantins	Palmas (256,5); Araguaína (250); Guaraí (78)	Ambulâncias
Campos Lindos	Tocantins	Araguaína (243,8); Balsas (Maranhão) (134)	Ambulâncias e UTI móvel (regulação)

UTI: unidade de tratamento intensivo.

Fonte: elaboração própria.

As condições do transporte sanitário, aliadas à situação das estradas e à distância até a referência ou até a uma metrópole ou capital regional como definido pelo Instituto Brasileiro de Geografia e Estatística (IBGE) ^9^, dificultaram os encaminhamentos. A demora em obter o apoio logístico gerou necessidades de aquisições, viabilizadas com os recursos transferidos pelo Governo Federal. Um município rural remoto recebeu uma ambulância de doação, enquanto outros contrataram o serviço de remoção: “*tenho três ambulâncias...todas...simples, quando...solicitava UTI móvel... era um valor exorbitante...*” (gestor; município 2) (Quadro 2). Entre os municípios rurais remotos, as distâncias para os centros urbanos de maior porte variam, sendo maiores para os municípios de Terra Nova do Norte (621,8km) e Maués (612km) e ainda aumentam quando os encaminhamentos são dirigidos a outros locais (Quadro 2).

Na assistência farmacêutica e laboratorial, a demanda aumentou, o que exigiu novos insumos e serviços, e a escassez ocorreu em todos os municípios rurais remotos, mas o esforço da gestão contribuiu, “*...não faltava medicação... o prefeito... falou, o que você precisar... vamos comprar... o que você indicar, a gente vai dar um jeito de licitar e comprar, e foi assim que foi feito*” (profissional da ESF - médico/a; município 4). O consumo de oxigênio exigiu apoio logístico, várias viagens diárias para suprir as necessidades. Um município referiu produção local com usinas de oxigênio ativas, criadas anteriormente à pandemia.

Foi comum a afirmação dos entrevistados quanto às dificuldades estruturais disponíveis nos centros mais próximos, como também no apoio laboratorial e na regulação dos casos graves. Nessa dimensão, estão os maiores problemas para acolher e resolver as demandas dos usuários.

### Organização

A reorganização do sistema local de saúde considerou a equipe, a capacidade instalada, os recursos transferidos, as parcerias e os arranjos estabelecidos. Inicialmente, alguns municípios suspenderam o atendimento de rotina, focando nos casos suspeitos de COVID-19. Posteriormente, foi restabelecido um fluxo de prioridade para gestantes, crianças e os cadastrados nos programas da unidade de saúde. Os demais pontos da rede, quando existentes, também suspenderam o atendimento e redistribuíram-se os profissionais para as atividades de monitoramento e prevenção conduzidas pelas equipes das UBS.

Na rede de atenção local, a maioria dos municípios rurais remotos analisados dispunha de pelo menos um dos serviços: Núcleos de Apoio à Saúde da Família (NASF), Centros de Apoio Psicossocial (CAPS) ou Centros de Reabilitação (Tabela 2). Apenas um município contava com os três tipos de serviços, e cinco com nenhum desses serviços formalmente habilitados. Independentemente de estrutura física específica, todos contavam com fisioterapeuta e apenas um município não disponibilizava psicólogo no serviço público. Na pandemia, os “*profissionais do NASF, psicólogo, fisioterapeuta, odontólogo... ficaram na unidade...*” (coordenador de atenção básica; município 11); “*as psicólogas, fizeram trabalho de grupos, pessoas que tinham sido contaminadas... os fisioterapeutas na... reabilitação daqueles... que estiveram internados... o CAPS não deixou de funcionar...*” (coordenador de atenção básica; município 10). Alguns municípios ofereceram apoio psicológico aos profissionais devido ao estresse e sobrecarga de trabalho.

Nas comunidades rurais, os agentes comunitários de saúde (ACS) entregaram medicamentos nos domicílios às pessoas com COVID-19 e àqueles com doenças crônicas, “*cada zona rural, cada território tem seu agente comunitário, tem que..., levar informação, buscar, monitorar... entra em contato...*” (gestor; município 9). Os ACS solicitavam ao serviço de referência o transporte sanitário e o atendimento aos sintomáticos de COVID-19 e acompanhavam os casos confirmados.

A separação entre atendimento de rotina e de sintomáticos respiratórios foi comum em todos os municípios rurais remotos, apesar das diferenças na capacidade instalada e na escassez de profissionais. Os centros de COVID-19 realizavam triagem, observação, acompanhamento, tratamento e estabilização até a remoção para a referência, outros utilizaram instalações de outros setores para triagem.

Naqueles poucos municípios rurais remotos que dispunham de unidade com leitos de internação, os locais de atendimento foram reorganizados para assistência de casos moderados ou graves, “*...lá no hospital também foi criada uma ala somente para COVID... com oxigênio instalado para todos os leitos... tem uma sala, semi UTI, faz os primeiros atendimentos para... aguardar essa vaga*” (coordenador de atenção básica/vigilância epidemiológica; município 8).

Com limitações, esses locais ofereceram segurança aos familiares, mas isso gerou estresse e tensão pela falta de estrutura e equipamentos que os casos requeriam “*...óbito graças a Deus a gente não passou essa situação... acontecer por falta de atendimento, mais vagas... era angustiante,... vivenciar corrida, todos os profissionais atrás das vagas...a gente se colocava na pele do médico... a preocupação era muita*” (coordenador de atenção básica; município 3).

Outro município do Mato Grosso afirmou que o hospital público de referência não ampliou o número de leitos na pandemia e ocorreu superlotação, “*as filas estavam grandes e necessitava dar uma resposta, mas... houve quatro óbitos esperando vaga em UTI. A gente ficava de mãos atadas, sem suporte*” (gestor e assistente social; município 4). Nesse município, para prover assistência aos casos não atendidos na referência regional, a gestão alugou um hospital privado desativado e, com limitações, assegurou o atendimento, mas tensionou o trabalho dos profissionais, que foram além da capacidade estrutural.

Os arranjos locais também foram viabilizados junto ao setor privado, devido ao aumento expressivo da demanda por internações. Em Tocantins, o estado e os municípios instalaram o hospital de campanha; em Mato Grosso, liderados pelos prefeitos e vereadores, o estado instalou leitos de UTI em hospitais do setor privado. Esses arranjos dependiam da maior ou menor presença estadual. A insuficiência de leitos gerou dificuldades para acolher e aguardar a vaga para encaminhamento até o centro de referência “*...poderia ter ampliado leitos e não teve...*” (profissional da ESF/coordenador de atenção básica - enfermeiro/a; município 2).

Os encaminhamentos para as regiões ou macrorregiões eram regulados pelo estado. A qualidade de alguns serviços foi questionada pelos usuários, “*um amigo... médico se referiu ao hospital regional* [de referência]*, no auge da pandemia, como um matadouro*” (coordenador de vigilância epidemiológica/representante do CMS; município 4). As dificuldades enfrentadas mostraram que as regiões precisam de investimentos: “*é muito difícil para a região, a estrutura, deixa muito a desejar, falta médico, profissionais, que atenda a média e a alta complexidade,... estou a setenta e oito quilômetros* [referência mais próxima]*.... e a duzentos e cinquenta de* [outra referência]*..., quase nada se resolve* [hospital de referência mais próximo]*, o que eu não resolvo aqui, pode se dizer que eu não resolvo lá*” (gestor; município 16).

Nessa dimensão, identificaram-se os esforços dos gestores e profissionais para organizar o atendimento dos sintomáticos respiratórios e dos usuários acompanhados pelas unidades de saúde antes da COVID-19. Foi comum a afirmação de pouca participação do estado na reorganização dos serviços da rede de apoio regionalizada e no transporte aéreo, gerando dificuldades no encaminhamento das demandas.

O Quadro 3 sintetiza os principais achados, por dimensão, categorias de análise e opiniões dos entrevistados.

**Quadro 3:** Síntese dos resultados da pesquisa por categorias de análise das dimensões da política, da estrutura e da organização dos serviços, com afirmações dos entrevistados dos municípios rurais remotos selecionados. Brasil, 2022.

CATEGORIAS DE ANÁLISE	SÍNTESE DOS ACHADOS DA PESQUISA	AFIRMAÇÕES DOS ENTREVISTADOS
POLÍTICA		
Plano de contingência	Modelo de gestão participativo; plano como instrumento de condução; experiência em planejamento e antecipação das medidas; apoio à gestão municipal pelo nível regional	“*Nessa reunião, alertava gestores e profissionais de saúde sobre medidas que deveriam ser tomadas a partir daquele dia*” (coordenador da atenção básica; município 10) “*...montar o plano, quais ações executar e colocar em prática foi uma das partes mais difícil; a gente teve apoio*” (coordenador da atenção básica/vigilância em saúde; município 16)
Comitê de enfrentamento	Criação e funcionamento do comitê com outros setores; comitê de natureza deliberativa, consultiva e condutor do plano; conflitos entre representantes; participação sócial reduzida	“*...a gente começou a se reunir, conversava sobre as decisões que ia tomar, só que as coisas foram acontecendo muito rápido*” (profissional da ESF - enfermeiro/a; município 10) “*não sei quem faz parte, não foi chamado o conselho*” (representante do CMS; município 7) “*a gente verificou a necessidade da Polícia Militar, solicitamos a Secretaria de Educação, Meio Ambiente, Tributos*” (gestor; município 10)
Orientação por decretos	Adequação dos decretos estaduais e federais à realidade do municípios rurais remotos; APS fortalecida à vigilância e educação em saúde; predominância de orientação à população; dificuldade em fazer cumprir os decretos - bares, festas e comerciantes	“*...estávamos numa situação em que se todo mundo não vestisse a camisa junto, todo mundo ia afundar junto...*” (profissional da ESF - enfermeiro/a; município 9) “*é difícil,... principalmente o público jovem*” (gestor; município 4) “*Tínhamos muita resistência... a gente entendia... era a reação das pessoas... desesperadas, um misto de confusão, um caos, uma situação que a gente nunca imaginou que veria*” (profissional da ESF - enfermeiro/a; município 9) “*...eles perceberam que se não se adequassem o comércio não poderia abrir...*” (profissional da ESF - enfermeiro/a; município 9)
Articulação entre níveis de governo	Recursos transferidos para aumentar a capacidade instalada; consórcios intermunicipais e ampliação do escopo de serviços; apoio técnico das instâncias regionais; articulação interfederativa à transferência de recursos financeiros e materiais	“*Ele* [deputado] *conseguiu também outros recursos para serem utilizados, no momento mais grave da pandemia...*” (gestor; município 10) “*...para você ter uma noção, o meu município tinha três tomografias mensais, então não dá para nada, então a gente comprava a maioria delas via consórcio*” (gestor; município 2) “*Do estado nós tivemos suporte, acredito que poderia ter mais,... não foi um suporte como a gente esperava da rede estadual*” (coordenador da atenção básica; município 10)
ESTRUTURA		
Insumos e serviços	Aquisição de insumos e equipamento básicos; custo elevado exames e insumos; dificuldade para obter insumos mesmo dispondo recursos financeiros; compra de serviços do setor privado; ampliação do escopo de serviços por meio de consórcios; apoio logístico insuficiente: transporte e volume	“*Sim, comprou tanto com recurso próprio, com os incrementos das emendas parlamentares,... recursos COVID..., que era para custeio... O consórcio ajudou na compra de EPI*” (gestor; município 2) “...*a gente buscava, todo dia* [oxigênio]*, saía daqui duas horas da manhã, às vezes dez horas da manhã de novo tinha que ir para buscar, graças a Deus..., ninguém ficou sem, mas era uma correria imensa para poder conseguir suprir tudo*” (gestor; município 4) “*Teve um aumento muito grande dos injetáveis..., com soro, teve, coisas que o município não tinha esse gasto*” (gestor; município 2) “*Sim, tem o consórcio e... as pactuações, que são feitas*” (assistente social; município 4)
Capacidade instalada ambulatorial, hospitalar e de leitos de UTI	Profissionais de saúde em número reduzido, dificuldade de fixação de recursos humanos; leitos hospitalares e de UTI inexistentes ou em pequena quantidade; insumos e equipamentos hospitalares e laboratoriais reduzidos; transporte hospitalar escasso	“*O hospital presta serviço para a prefeitura; vendo certinho os locais, onde que ia ser a ala, o que precisava comprar, o que é, que materiais o hospital ia precisar, então assim, a parceria é bem grande mesmo, hospital e Secretaria da Saúde. O laboratório nosso era terceirizado; em Pontes e Lacerda. O município comprou serviço fora do ofertado do estado, quando acabou o dinheiro, acabou a alegria, aí infelizmente muitos pacientes ficaram à mercê. O que ele comprou, tomografia, o que mais, nós compramos tomografia*” (profissional da ESF - enfermeiro/a; município 8)
Acesso à UBS povos tradicionais	SMS disponibilizou transporte para acesso à unidade de referência urbana e rural	“*Temos comunidade quilombola, tivemos um caso confirmado, a gente faz a assistência, o médico vai fazer a visita*” (profissional da ESF - enfermeiro/a; município 9) “*A gente chama de comunidade polo onde fica o posto de saúde, se tiver algum sinal, sintoma procurava esse posto, se o técnico avaliasse que poderia ser algo sugestivo a covid removia*” (coordenador de atenção básica; município 10)
SADT e assistência farmacêutica	Prescrição/Entrega domiciliar de medicamentos de uso contínuo; ausência de pactuação interestadual; contratação de serviços laboratoriais/imagem	“...*o remédio, o agente mesmo levava para a pessoa... de diabetes, hipertensão?... da zona rural sim*” (coordenador da atenção básica; município 11) “*A gente precisou muito... exame de tomografia, raio-X,... não tinha laboratório no município, quando precisava tinha que encaminhar... para o hospital de referência mais próximo...*” (coordenador da atenção básica; município 16) *“...a gente comprava a maioria delas* [TC] *via consórcio*” (gestor; município 2)
Transporte para fora do município rural remoto	Transporte sanitário reduzido/inadequado, com necessidade fluvial/aérea; custo elevado para contratar UTI móvel, ausente na região; profissionais sobrecarregados para acompanhar os pacientes	“...*a UTI móvel estava aqui, e ela saiu daqui entubada*” (gestor; município 16) “*Teve também um carro, uma ambulância, que era tomografia toda hora de paciente, toda hora*” (assistente social; município 4) “*...aconteceu até de contratar UTI móvel...*” (coordenador da atenção básica; município 2) “*Tomografia mais próxima... dá 90km, então era ambulância indo duas, três vezes para lá e tinha que levar só um paciente*” (assistente social; município 4) “*...a gente conseguiu consertar na época as três ambulâncias, mas agora acabaram duas, só ficou uma aqui*” (gestor; município 11)
ORGANIZAÇÃO		
Fluxo inicial na pandemia e atendimento de rotina	Rotina de atendimento com prioridade à COVID-19; cuidados no isolamento; agendamento diferenciado, estrutura separada e equipes distintas; manutenção de canais de comunicação com a população	“*Reduzimos o fluxo da unidade, os serviços foram suspensos., e depois. entendemos. que teríamos que retomar*” (profissional da ESF/coordenador da atenção básica - enfermeiro/a; município 3) “*No início foram mais usadas por meio de telefone, telecomunicação criou uma rotina priorizando os atendimentos... foi organização da UBS para não parar o atendimento*” (profissional da ESF - enfermeiro/a; município 16) “...*não queriam ir nas unidades, não queriam sair de casa, hoje está aparecendo muitas pessoas com diabetes e hipertensão, ansiedade... as psicólogas não estão dando conta de atendimento*” (gestor; município 4)
Unidade de referência local para COVID-19	Reorganização diferenciada; instalação de unidade de referência COVID-19; pontos de contato rural (postos) e transporte (rodoviário ou fluvial)	“*Com a criação do centro do COVID, houve essa separação,... o paciente que ia ser internado já saía com a prescrição, a ambulância pegava e levava até o setor de internamento...*” (profissional da ESF - médico/a; município 7) “...*pegamos uma UBS... para funcionar 24 horas como um pronto socorro, só para os sintomáticos respiratórios...*” (gestor; município 10) “...*tivemos que alugar um hospital 24 horas*” (gestor; município 4) “*Os pacientes* [interior] *que tinham condições, vinham no carro próprio, caso fosse emergência, a ambulância busca*” (coordenador da atenção básica; município 2)
Articulação com a rede	Rede local limitada à APS; coleta laboratorial local; equipes de NASF/CAPS no atendimento à COVID-19; fisioterapeutas e psicólogos	“*O NASF nos apoiou muito na ESF*” (coordenador da atenção básica; município 10) “*Fisioterapia é muito contato, então não tinha como estar fazendo, a não ser que fosse um caso grave*” (profissional da ESF - enfermeiro/a; município 14) “*O NASF participou na reabilitação, após o paciente ter COVID, precisava de reabilitação pulmonar, fisioterapia*” (coordenador da atenção básica; município 16)
Regulação e fluxo de referência	Baixa articulação interfederativa; vazios assistenciais público-privados; cuidado gerenciado para além da gravidade e da rede local; atuação além das atribuições/autossuficiência da rede e dos recursos humanos/financeiros; regulação e deslocamento dificultadas - logística	“*A regulação em si era ruim... às vezes faltava vaga*” (profissional da ESF - enfermeiro/a; muncicípio 14) “*A regulação; tivemos um período bem crítico*” (profissional da ESF/coordenador da atenção básica - enfermeiro/a; município 3) “*O médico entubou ela dentro da unidade,... a paciente graças a Deus está bem, fizeram até agradecimentos nas redes sociais...*” (gestor; município 16) “*O estado falhou porque poderia ter melhorado..., ter ampliado leitos... principalmente municípios mais remotos como é o nosso, a gente fica meio à mercê, mais dificultoso*” (profissional da ESF/coordenador da atenção básica - enfermeiro/a; município 3) “...*chegamos a ficar com o paciente aguardando três, quatro dias,... nós tivemos de fila de espera...*” (gestor; município 10) “*Via aérea era bancada pelo município, não tinha quase nenhum apoio do Estado em relação a essas transferências, mas da COVID para cá, melhorou 300%, hoje o Estado conta com o serviço*” )gestor; município 10)

APS: atenção primária à saúde; CAPS: Centros de Apoio Psicossocial; CMS: Conselho Municipal de Saúde; EPI: equipamentos de proteção individual; ESF: Estratégia Saúde da Família; NASF: Núcleo de Apoio à Saúde da Família; SADT: Serviço de Apoio Diagnóstico Terapêutico; SMS: Secretaria Municipal de Saúde; UBS: unidade básica de saúde; UTI: unidade de tratamento intensivo; TC: tomografia computadorizada

## Discussão

Situados em diferentes estados e com suas singularidades, os municípios rurais remotos analisados responderam aos desafios da pandemia com o protagonismo da APS ^1^. De forma diferenciada, as necessidades em saúde foram parcialmente respondidas em tempo oportuno, na perspectiva da integralidade do cuidado. Apresentam dificuldades de acesso aos grandes centros, com fragilidades e irregularidades no transporte sanitário adequado e no uso de tecnologias de informação que geraram insegurança aos usuários e aos profissionais ^8^ ^,^ ^11^ ^,^ ^24^. Entre esses municípios, há desigualdades socioespaciais que combinam abundância e escassez, expressas pelo PIB *per capita* e índice de Gini. Revelam formas de acumulação desigual que, associadas aos baixos investimentos públicos, geram disfunções sociais, acesso diferenciado aos serviços de saúde e afetam as condições de vida e de saúde da população ^6^ ^,^ ^25^ ^,^ ^26^.

Os resultados deste estudo de casos múltiplos não garantem a representatividade do conjunto dos municípios rurais remotos; porém, apresentam os impasses comuns enfrentados durante a pandemia da COVID-19. As entrevistas virtuais buscaram captar a condição do sistema de saúde no contexto rural remoto. Apresentaram limitações técnicas (interrupções; somente áudio em alguns momentos) e de análise da linguagem não verbal que é mais ampla quando a entrevista é presencial. No entanto, a experiência dos entrevistadores e a interação com eles foram bastante positivas, dentro do cenário possível.

Esses municípios têm em comum um sistema de saúde restrito à APS, com pontos de atenção na sede e alguns no interior ^1^ ^,^ ^11^. Durante a pandemia, a resposta e o cuidado às necessidades variaram, disponibilizaram transporte sanitário rodoviário e fluvial, criaram estratégias que contemplaram as especificidades para equalizar a assistência à saúde e não restringir o acesso ao cuidado oferecido nesse nível. Com muitas limitações, e nem sempre com o apoio dos demais entes interfederativos, o empenho da gestão e dos profissionais contribuiu para facilitar o acesso à rede de serviços locorregional, reorganizar a APS e estabelecer a comunicação, de forma semelhante a outros países ^16^ ^,^ ^27^ ^,^ ^28^ ^,^ ^29^.

Nas unidades de APS, ocorreram mudanças na rotina de cuidado, inicialmente muitas foram fechadas e reabertas em seguida, priorizando o atendimento dos sintomáticos de COVID-19. Nas diferentes ondas da pandemia, iniciadas em março de 2020 que se prolongaram em alerta até meados de 2022, as equipes acolheram as demandas, mantiveram a rotina seletiva de atendimento, monitoraram os inscritos nos programas e os casos de COVID-19 ^10^ ^,^ ^30^ e não interromperam a comunicação, responsabilizando-se pela população adscrita ao território ^30^.

A vulnerabilidade nos municípios rurais remotos ocorre pela inacessibilidade geográfica, escassez de profissionais e serviços de saúde, tempo de deslocamento, condições de transporte sanitário, financiamento insuficiente, distribuição e uso dos recursos públicos. Esses achados confirmam que as populações rurais utilizam menos os serviços de saúde ^3^ ^,^ ^31^, têm barreiras de acesso aos demais níveis de complexidade e se assemelham ao contexto de populações rurais de outros países, como Canadá e Austrália ^4^ ^,^ ^23^ ^,^ ^27^. Essas limitações interferem nas práticas de saúde, no acesso, na integralidade e no cuidado ofertado.

Estudo sobre as regiões do SUS advertiu que parte significativa apresenta insuficiência e escassez de serviços nas referências regionais para prover o atendimento aos acometidos pela COVID-19 ^6^ ^,^ ^32^ ^,^ ^33^. Os CIS e os hospitais de campanha instalados em alguns estados constituíram arranjos interfederativos para realizar a aquisição de insumos, contratualizar serviços de imagens e laboratoriais, internações hospitalares e leitos de UTI ^2^ ^,^ ^32^ ^,^ ^34^. Estabelecidos na emergência da pandemia, minimizaram as iniquidades e melhoraram o acesso aos demais níveis de complexidade ^1^ ^,^ ^23^ ^,^ ^28^ ^,^ ^29^. No entanto, são considerados frágeis e podem aumentar as desigualdades em saúde se as políticas públicas não contemplarem a realidade dos municípios rurais remotos, se não houver coordenação e reconhecimento por parte do Estado ^32^ das condições de vulnerabilidade a que estão expostos.

Na pandemia, tornaram-se evidentes os esforços para prover o acesso e o cuidado integral a partir da APS, aproximando-se de seu papel na coordenação do cuidado. Denotam diferentes contextos, capacidades desiguais ^3^, respostas diferentes para situações semelhantes que traduzem as condições desiguais para o cuidado. Requerem, para além do apoio técnico regional: fortalecer as instâncias regionais; contemplar as regiões fronteiriças ^33^; compartilhar os recursos interfederativos; e implementar políticas públicas de Estado. As especificidades dos municípios rurais remotos e suas regiões, há muito negligenciadas, devem ser contempladas, de modo a aprimorar o sistema de saúde locorregional e a capacidade de gestão do SUS, com o foco na equidade em saúde ^35^.

A crise da pandemia acentuou os problemas já enfrentados pelos municípios rurais remotos e, apesar da sua limitada capacidade, reorganizou e fortaleceu a APS ^36^ ^,^ ^37^ e o sistema local de saúde. No entanto, a APS requer investimentos e comprometimento dos entes federativos ^1^ para dar sustentabilidade ao fortalecimento observado na pandemia.

As especificidades dos municípios rurais remotos ^1^ ^,^ ^8^ ^,^ ^10^ ^,^ ^11^ foram reiteradas e, para além das destacadas, ainda há necessidades de esclarecer os papéis interfederativos e de aprimorar os mecanismos de cooperação intergovenamental. Os arranjos de governança podem fortalecer os espaços locorregionais, validar a proteção à saúde e prover serviços que facilitem o acesso equitativo ao cuidado em saúde, de forma contínua e integral, com qualidade e acesso oportuno ^2^ ^,^ ^8^ ^,^ ^35^ ^,^ ^38^.

Foram frequentes os relatos de conflitos e de dificuldades, mas também das iniciativas e empenho dos municípios rurais remotos para resolver as demandas durante a pandemia. Além do avanço estrutural do sistema de saúde, necessitam do apoio técnico e protagonismo do estado na condução da política de saúde. Os resultados sugerem intervenções interfederativas que abarcam as dimensões exploradas, para, de forma integrada e estratégica, fortalecer e contribuir para reduzir as fragilidades do sistema de saúde, potencializadas pelas barreiras de acesso e acentuadas durante a pandemia da COVID-19.

A abordagem multidimensional identificou autonomia, mas também interdependências entre os condicionantes políticos, estruturais e organizacionais na reorganização do sistema de saúde locorregional. O conjunto das informações obtidas dos atores inseridos no sistema municipal de saúde demonstrou as experiências comuns e as especificidades quanto aos problemas mais recorrentes enfrentados pelos municípios rurais remotos.

## Considerações finais

Partindo de importantes limitações, os municípios rurais remotos analisados responderam aos desafios enfrentados na pandemia no período de 2020 a 2022. Seguindo as medidas de contingenciamento orientadas pelos decretos dos governos federal e estadual, constituíram os comitês, mas poucos elaboraram o plano de contingenciamento. Todos receberam recursos financeiros fundo a fundo. As dificuldades enfrentadas variaram entre os municípios rurais remotos, expressas por: suas características socioespaciais, indicadores econômicos e sociais, distâncias até os centros de referência, escassez de serviços do primeiro nível de atenção, transporte sanitário inadequado e acesso inoportuno aos serviços da rede de atenção regionalizada.

Independentemente do escore alcançado na análise documental, reorganizaram o sistema local com limitações, e a APS aproximou-se de seu papel na coordenação do cuidado. As equipes trabalharam com determinação, preservaram a comunicação com a população, buscaram alternativas para acolher e atender às demandas, alteraram o funcionamento das unidades de saúde e, de forma responsável, não interromperam a rotina de cuidado realizando o monitoramento dos casos de COVID-19 e dos inscritos nos programas.

Os fluxos regionais foram definidos pelo estado, mas as lacunas assistenciais da rede de serviços secundários, terciários e de apoio diagnóstico, impediram que suas necessidades fossem atendidas em tempo oportuno. De maneira prudente e sem capacitação específica, os profissionais ultrapassaram os limites de suas atribuições para prestar o cuidado necessário e aguardar o encaminhamento aos demais níveis de complexidade.

Este estudo mostrou que a disponibilidade, a localização e a entrada na rede de serviços influenciam a qualidade do cuidado em tempo hábil e integral. A rede de atenção locorregional requer comprometimento dos entes interfederativos com investimentos na estrutura física, equipamentos, capacitação das equipes e apoio logístico. É uma situação complexa que reitera o subfinanciamento crônico do SUS, os vazios assistenciais da rede de atenção, e requer fortalecimento das relações interfederativas. O empenho, o esforço e a integração das equipes resultaram em experiências exitosas; mas a provisão equitativa e resolutiva do sistema local de saúde nos municípios rurais remotos, em resposta a crises sanitárias, implica na rearticulação interfederativa, na formulação e implementação de políticas públicas, que são elementos imprescindíveis à superação de desafios e, ao mesmo tempo, põem em relevo a vitalidade do SUS.
